# *Haloferax volcanii*: a versatile model for studying archaeal biology

**DOI:** 10.1128/jb.00062-25

**Published:** 2025-05-14

**Authors:** Mechthild Pohlschroder, Stefan Schulze, Friedhelm Pfeiffer, Yirui Hong

**Affiliations:** 1 Department of Biology, University of Pennsylvania 6572, Philadelphia, Pennsylvania, USA; 2 Thomas H. Gosnell School of Life Sciences, College of Science, Rochester Institute of Technology 173221 , Rochester, New York, USA,; 3 Computational Systems Biochemistry, Max Planck Institute of Biochemistry 28311, Martinsried, Bavaria, Germany; University of Florida, Gainesville, Florida, USA

**Keywords:** archaea, model organisms, *Haloferax volcanii*, molecular biology, history, genetics, biotechnology, cell biology, education, biochemistry

## Abstract

Archaea, once thought limited to extreme environments, are now recognized as ubiquitous and fundamental players in global ecosystems. While morphologically similar to bacteria, they are a distinct domain of life and are evolutionarily closer to eukaryotes. The development of model archaeal systems has facilitated studies that have underscored unique physiological, biochemical, and genetic characteristics of archaea. *Haloferax volcanii* stands out as a model archaeon due to its ease of culturing, ability to grow on defined media, amenability to genetic and biochemical methods, as well as the support from a highly collaborative community. This haloarchaeon has been instrumental in exploring diverse aspects of archaeal biology, ranging from polyploidy, replication origins, and post-translational modifications to cell surface biogenesis, metabolism, and adaptation to high-salt environments. The extensive use of *Hfx. volcanii* further catalyzed the development of new technologies and databases, facilitating discovery-driven research that offers significant implications for biotechnology, biomedicine, and core biological questions.

## INTRODUCTION

Archaea are key members of virtually all ecosystems (1), playing crucial roles in global nitrogen and carbon cycles (2), and having extensive applications in biotechnology and bioremediation (3–5). Beyond their ecological significance, archaea also impact agriculture (6) and human health (7), as they are integral members of plant, animal, and human microbiomes (8).

Research in archaeal model systems has offered valuable insights into archaeal biology, as well as the cell biology and evolution of all domains of life. Archaea share certain biological characteristics with bacteria, including the chemotaxis system (9), type IV pili (cell surface structures required for surface attachment and biofilm formation) (10), certain surface-anchoring pathways (11), and cytoskeletal proteins (12, 13). Yet, they also exhibit unique features that distinguish them as a separate domain, such as cytoplasmic membrane lipids with distinct stereochemistry and compositions (14), a cell wall lacking peptidoglycan (15), and archaella, rotating motility appendages that mediate swimming motility (16, 17). Importantly, archaea share biological features with eukaryotes, including genetic information processing and protein trafficking, reflecting a closer evolutionary relationship between archaea and eukaryotes (18–20). Being comparatively simple, several fundamental eukaryotic biological processes were initially resolved using the related archaeal systems. Examples for such insights include structural studies of ribosomes (21), DNA replication (22), secretory (Sec)-dependent protein translocation (23), and chaperonins (24), as well as functional analyses into proteasomes (25).

Four archaeal groups have emerged for the development of model systems, largely due to their suitability for genetic manipulations: methanogens, thermophiles, thermoacidophiles, and haloarchaea (26, 27). Methanogens (e.g., *Methanococcus* and *Methanosarcina*) have received increasing attention due to their role in producing methane, a potent driver for global warming (28). Thermophiles (e.g., *Thermococcus* and *Pyrococcus*) have been studied for a range of metabolic and enzymatic functions (29), and the thermoacidophiles of the genus *Sulfolobus* are the only archaeal model system belonging to the phylum Thermoprotei, previously Crenarchaeota, while all others are members of Methanobacteriota, previously Euryarchaeota. Compared to the high temperatures and/or anaerobic growth conditions of the aforementioned model species, haloarchaea are easy to culture as they grow in oxic environments at moderate temperatures. They can be divided into moderate (0.5–2.5 M NaCl) and extreme (>2.5 M NaCl) halophiles, and a representative of each group, *Haloferax volcanii* and *Halobacterium salinarum*, respectively, has been developed into prominent model systems for archaeal research.

In 1940, Benjamin Elzar-Volcani showed in his PhD thesis that the Dead Sea is full of microbial life (30). To honor his discovery, an organism isolated from the mud of the Dead Sea in 1975 was named after Dr. Volcani as *Halobacterium volcanii* (31). It was believed to be closely related to *Halobacterium salinarum*, an extreme halophile first isolated from cured codfish in 1922 (32) and 12 years later from cow and buffalo hides (33). However, the moderate salt requirements of *Halobacterium volcanii*, along with phenotypic differences such as a distinct lipid composition, led to its reclassification as *Haloferax volcanii*, the type species of the newly established genus *Haloferax* (34). Conversely, several extreme halophiles that were initially considered distinct species (*salinarum*, *cutirubrum*, and *halobium*) were shown to be so similar that they were reclassified as a single species, *Hbt. salinarum* (35).

**Fig 1:**
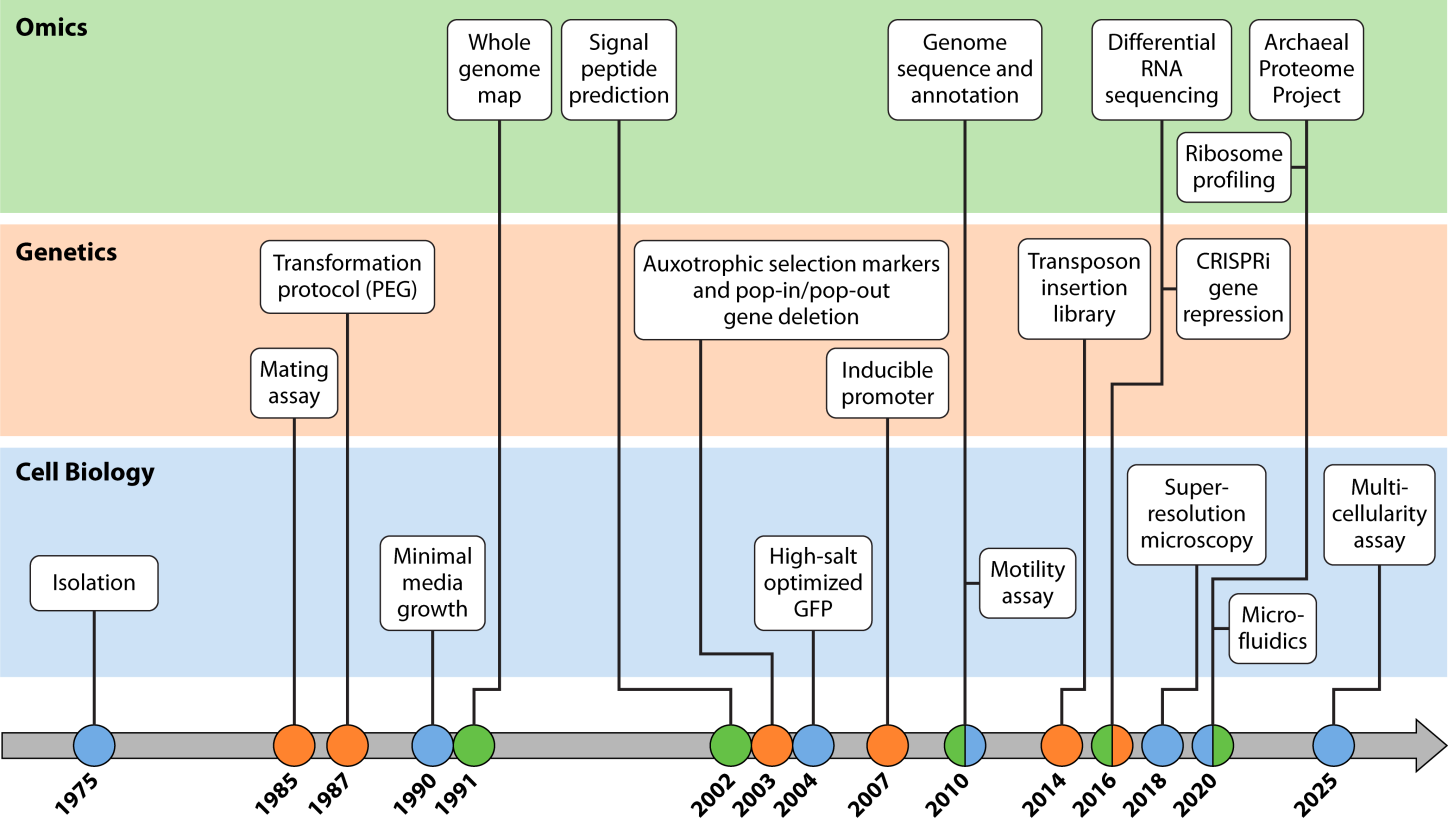
A timeline of methodological milestones that established *Haloferax volcanii* as a key model organism to advance the biological characterization of Archaea. Cell biology advances in *Hfx. volcanii* include isolating *Hfx. volcanii* from the Dead Sea (31), establishing efficient growth of *Hfx. volcanii* in minimal media (41), enabling the usage of green fluorescent protein (GFP) under high-salt conditions (43), establishing conditions under which *Hfx. volcanii* shows motility (44), adapting super-resolution microscopy to image individual molecules (45), using microfluidics to build mother machines (46), and developing a multicellularity assay that results in tissue-like cell complexes (47). Genetic advances played major roles in establishing *Hfx. volcanii* as a model archaeon, including developing the first genetic information transfer assay for an archaeon (the mating assay) (48), adapting a transformation protocol to *Hfx. volcanii*, making this the first archaeon transformed with a plasmid (49), developing auxotrophic selection markers and the pop-in/pop-out method to generate marker-less deletion strains (50), adopting an inducible promoter for regulated gene expression/silencing (51), as well as generating a whole-genome transposon insertion library (52) and adapting a method to silence archaeal genes: clustered regularly interspaced short palindromic repeats interference (CRISPRi)(53). Early omics approaches such as publishing a detailed map of the whole genome, including a pan-genome set of cosmid clones (54), and predicting signal peptides for Sec, twin-arginine transport, and Pil/Fla pathways (55), were followed by more advanced system-wide analyses, allowing researchers to gain insights at the molecular level. These include publishing the complete genome sequence and annotation of the *Hfx. volcanii* DS2 strain (56), establishing differential RNA sequencing to identify transcriptional start sites (57), adapting ribosome profiling for *Hfx. volcanii* (58), and combining information about its proteome in the Archaeal Proteome Project (59). PEG, polyethylene glycol.

Many key biological observations were made in both *Hbt. salinarum* and *Hfx. volcanii*, such as the identification and characterization of archaeal glycoproteins (36, 37) and the confirmation of polyploidy (38). However, the two species also exhibit distinct properties. For example, research on bacteriorhodopsin and other retinal proteins is primarily performed using *Hbt. salinarum* (39, 40) as retinal proteins are absent in *Hfx. volcanii*. On the other hand, *Hfx. volcanii* offers several advantages as a model organism, including its moderate salt requirements, nutritional simplicity, and better genetic stability (27, 41, 42). While both species, as well as other model archaea, are widely used and have significantly advanced the field of archaeal biology, this review will focus on *Hfx. volcanii* due to the breadth of available techniques for this species that facilitated a multitude of fundamental studies to understand cellular functions and pathways in archaea (Fig. 1). Here, we will outline key methods that comprise the toolbox for *Hfx. volcanii* and establish it as a versatile model system. In addition, we will highlight major biological insights obtained through studies in *Hfx. volcanii* using these tools, which often have broad implications for research in other archaea, bacteria, and eukaryotes.

## ESTABLISHING *HFX. VOLCANII* AS A MODEL ARCHAEON

A key factor that established *Haloferax volcanii* as the leading model organism is its notable genetic tractability. It has a well-established transformation protocol, a rich collection of selectable markers and cloning vectors, and a streamlined gene knockout system. These tools, along with others compiled in the *Halohandbook* (60), were crucial in advancing *Hfx. volcanii* studies (Table 1). Here, we provide a short review of these early achievements and how these led to the establishment of *Hfx. volcanii* as a model archaeon.

**TABLE 1:** Representative tools for *Hfx. volcanii*

Application	Description
**Growth**	
Rich media	MGM (60) Hv-YPC (61)
Semi-defined media	Hv-Ca (61) Hv-Cab (62)
Minimal media	CDM (41) Hv-Min (61)
**Genetics/genomics**	
Genome	Genome sequence and annotation of the DS2 strain (56) Resequencing of several engineered lab strains (63)
Transformation	Polyethylene glycol-based transformation (49)
Inducible promoters	PtnaA, tryptophan induced promoter (51, 64) Pxyl- and xylose-induced promoter (65)
Constitutive promoters	Pfdx, ferredoxin promoter (66) Psyn, a synthetic promoter (67) P250, the first 250 bp of the *xacEA* promoter (P250) (65)
Tandem expression vectors	Tandem expression plasmids containing the two fusion genes expressed from the same PtnaA promoter (68)
Selectable markers	Mevinolin resistance (69) Simvastatin resistance (70) Novobiocin resistance (71) Orotate phosphoribosyl transferase gene, *pyrE2*, for uracil auxotrophic strains (50) Tryptophan synthase gene, *trpA*, for tryptophan auxotrophic strains (61) 3-Isopropylmalate dehydrogenase gene, *leuB*, for leucine auxotrophic strains (61) Dihydrofolate reductase gene, *hdrB*, for thymidine auxotrophic strains (61, 72)
Gene deletion	Pop-in/pop-out based on the DS70 strain (61) Pop-in/pop-out based on the WFD11 strain (50) A streamlined pop-in/pop-out protocol (73) A Gateway platform for gene deletion (74)
Transposon insertion mutant library	A collection of mutants, each containing a single genomic transposon insertion (52)
Genome architecture	Hi-C optimized for better resolution (75)
Transcription analysis	Differential RNA-Seq (dRNA-Seq) (57) Comparative strand-specific small RNA sequencing (76) Mixed RNA-Seq (dRNA-Seq and RNA-Seq combined) (77) rRNA removal (78) Nanopore-based RNA sequencing (79)
Translation	Ribosome profiling (58, 80)
**Protein characterization**	
Signal peptide prediction	TatFind (55) FlaFind (81) TatLipo (82) SignalP 6.0 (83)
Proteomics	Label-free quantitative SWATH-MS (84) Small protein optimized MS analysis (80) Proteome turnover MS analysis (85) Multiplex quantitative SILAC (86)
Protein Purification	Cleavable and non-cleavable affinity tags, including 8xHis-tag, 6xHis-tag, Strep-tag, Twin-Strep-tag, FLAG-tag, 3xFLAG-tag, C-tag (87) Protein purification using *Hfx. volcanii* H1895 in a bioreactor (88)
Protein expression regulation	CRISPRi (53, 89) Synthetic theophylline-dependent translational riboswitches (90)
Reporters	Beta-galactosidase (91) mGFP6 (92) Bioluminescence (93) Dihydrofolate reductase (94) Arabinose dehydrogenase (94)
**Imaging**	
Live-cell microscopy	Single-molecule localization microscopy (95) Super-resolution 3D-SIM fluorescence microscopy (45)
Non-live-cell microscopy	Transmission electron microscopy (96) Scanning electron microscopy (97) Cryo-electron microscopy (98) Cryo-electron tomography (98)
**Database**	
Haloweb	A haloarchaeal genome database (99)
The Archaeal Proteome Project	A combined reanalysis of *Hfx, volcanii* proteomics data sets (59)
The Archaeal Clusters of Orthologous Genes	A curated database of clusters of genes within the archaeal domain with shared evolutionary history (100, 101)

The first challenge researchers grappled with was the development of an efficient transformation method. In 1985, Mevarech and Werczberger identified the first archaeal genetic transfer system in *Hfx. volcanii*, demonstrating that archaeal cells could exchange endogenous DNA (48). Two years later, Cline and Doolittle made a breakthrough in introducing exogenous DNA into archaeal cells. They achieved satisfactory efficiency in transforming halovirus DNA into *Hbt. salinarum* using a polyethylene glycol 600-mediated spheroplast transfection method (102). Building on this, Charlebois et al. (49) adapted this method for *Hfx. volcanii* and successfully transformed the endogenous plasmid, pHV2, into WFD11, a pHV2-cured *Hfx. volcanii* strain, marking the first successful plasmid transformation in an archaeal species.

Because archaea are naturally resistant to most bacterial antibiotics, novel selectable markers had to be developed for selecting desired transformants. Mevinolin, an inhibitor of eukaryotic HMG-CoA reductase, was shown to inhibit the HMG-CoA reductase in *Hbt. salinarum* and prevent cell growth (103). Spontaneously, mevinolin-resistant clones were observed, and the resistance gene was cloned into a derivative of pHV2 (103). The cloning vector was then converted to shuttle vectors suitable for replication and selection in both *Escherichia coli* and *Hfx. volcanii* (pWL101 and pWL102) (69). Similarly, selection systems based on the mevinolin derivative simvastatin (70) and the DNA gyrase inhibitor novobiocin (71, 104) were developed, greatly facilitating genetic manipulation in *Hfx. volcanii*.

A major breakthrough in the molecular cloning of *Hfx. volcanii* was the development of a gene knockout system by Bitan-Banin et al. (50). Their landmark study demonstrated that the *Hfx. volcanii pyrE2* gene encodes an orotate phosphoribosyl transferase and is essential for uridine biosynthesis. Strains lacking *pyrE2* require uracil supplementation to grow, while strains containing *pyrE2* are susceptible to 5-fluoroorotic acid, which is converted to the toxic 5-fluorouracil. Using this feature, Bitan-Banin et al. developed the pop-in/pop-out method for marker-free gene deletion. In this approach, sequences flanking the target gene are cloned into a non-replicative plasmid carrying *pyrE2*, which integrates into the chromosome of a WFD11-derived ∆*pyrE2* strain via homologous recombination (the “pop-in” step). Transformed cells are then selected in uracil-free media and are subsequently transferred to media containing 5-fluoroorotic acid and uracil to induce plasmid loss via intrachromosomal recombination (pop-out step). Removal of the plasmid generates either wild-type revertants or the desired deletion strain, which is further screened by PCR and confirmed through whole-genome sequencing.

As WFD11-derived strains exhibited plasmid instability and growth defect due to the use of ethidium bromide in its generation, the Dyall-Smith lab developed a new pHV2-cured strain, DS70, without using mutagens (70). The Allers lab adapted the gene knockout system for DS70-derived strains, which have been used for gene deletion in *Hfx. volcanii* since then (61). They also introduced additional selection systems based on leucine (*leuB*), tryptophan (*trpA*), and dihydrofolate reductase (*hdrB*). The resulting strains, especially H26 (Δ*pyrE2*), H53 (Δ*pyrE2*Δ*trpA*), and H98 (Δ*pyrE2*Δ*hdrB*), along with a series of complementary vectors, have become the core tools for the highly accessible genetic manipulation of *Hfx. volcanii*.

The full genome sequence of the *Hfx. volcanii* DS2 strain was published in 2010 through a collaborative effort among multiple laboratories (56). However, the data had been shared with the scientific community several years earlier to support and accelerate research. Genomic analyses revealed that the type strain contains a primary chromosome of 2.848 Mb, along with three smaller chromosomes—pHV1 (85 kb), pHV3 (438 kb), and pHV4 (636 kb)—as well as the pHV2 plasmid (6.4 kb). This publication filled the gap in omic data for this emerging model organism and laid the foundation for subsequent proteomics and transcriptomics studies.

Beyond the technical breakthroughs, a key factor in establishing *Hfx. volcanii* as a leading model organism is its highly collaborative research community. Urgent needs for new or improved methodologies are continually addressed by proactive labs and promptly shared within the community, often before publication. The spirit of intensive collaboration is perhaps most evident in joint projects. An early example is the *Halohandbook* (60), which is an extensive collection of methods available for haloarchaeal research contributed by 26 researchers worldwide and compiled by Mike Dyall-Smith. This collection included both published and unpublished protocols, and thereby its release significantly accelerated the progress of *Hfx. volcanii* studies. More recent initiatives extended beyond *Hfx. volcanii* to include other archaeal species, such as the book *Archaea: Methods and Protocols* (105), the protocol repository at ARCHAEA.bio, the Archaea Power Hour (a monthly virtual seminar series), the Archaeal Proteome Project (59), and the collaborative resequencing of several widely used archaeal model strains (63). These efforts continue this tradition of collaboration (106), further propelling the development of model archaea, including *Hfx. volcanii*, as a powerful tool for scientific discovery.

## UNRAVELING ARCHAEAL BIOLOGY WITH *HFX. VOLCANII*

**Fig 2:**
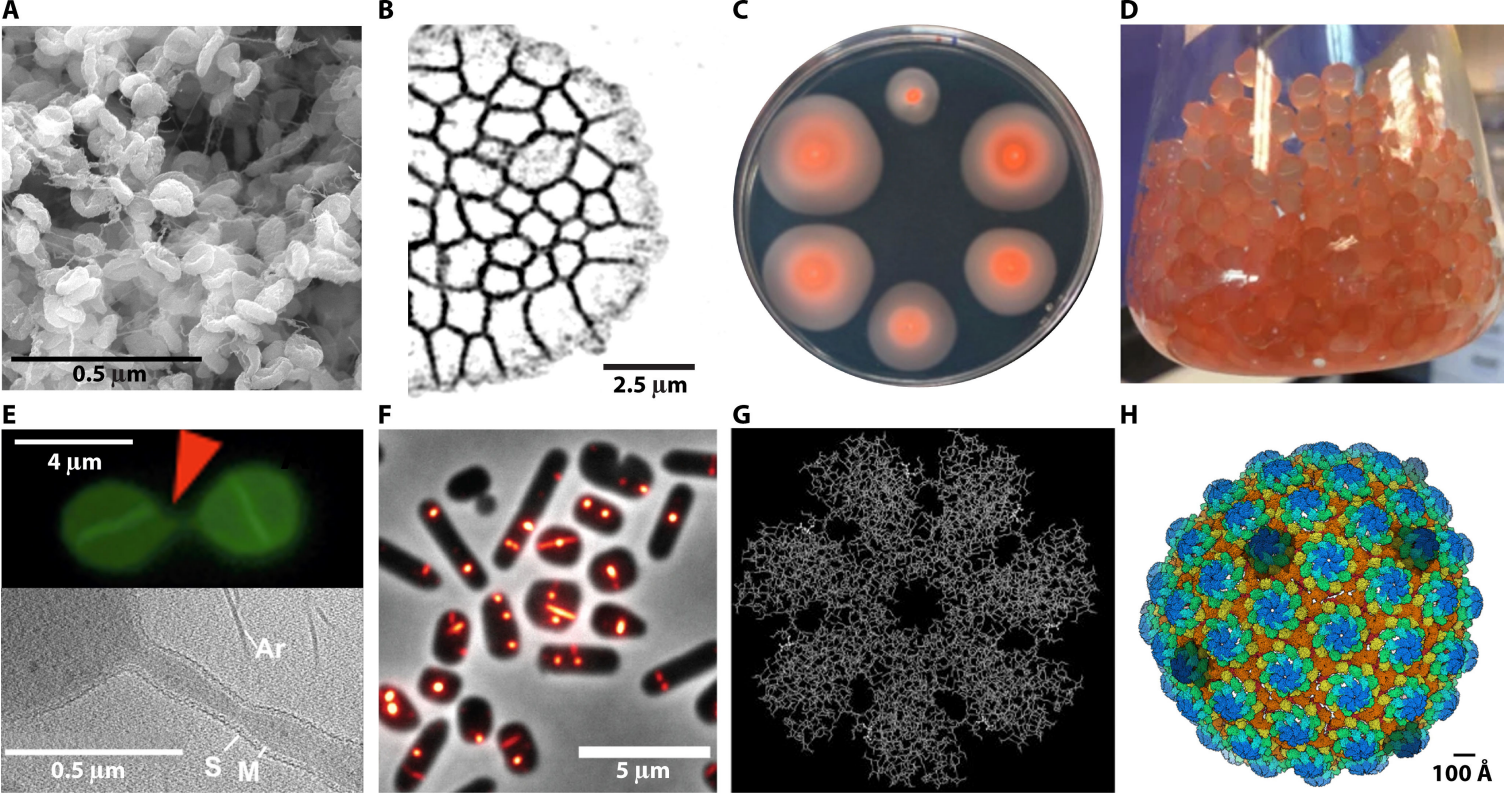
Biological insights and biotechnological advances in *Hfx. volcanii*. (**A**) Scanning electron microscopy of *Hfx. volcanii* biofilm grown on a nitrocellulose membrane. Courtesy of John Mallon and Arthur Charles-Orzsag. (**B**) The three-dimensional super resolution by optical reassignment microscopy images of *Hfx. volcanii* multicellular clone (courtesy of Alexandre Bisson). (**C**) Motility halos of *Hfx. volcanii* wild type (top center) and transposon insertion mutants (107). (**D**) *Hfx. volcanii* encapsulated within calcium alginate beads (67). (**E**) Formation of a cell–cell bridge in AlexaFluor488-labeled cells (top). A tomographic slice from a reconstructed tilt series of electron cryo-microscopy (bottom). The cytoplasmic membrane (M), the surface (S) layer (S), and archaella (Ar) are indicated (108). (**F**) Localization of green fluorescent protein-tagged volactin in mid-log phase *Hfx. volcanii* rod- and disk-shaped cells (109). (**G**) Structural model of the heptameric α1_7_-ring of *Hfx. volcanii* 20S core particles, the central catalytic part of the proteasome. Used with permission of John Wiley & Sons, Inc., from reference 110; permission conveyed through Copyright Clearance Center, Inc. (**H**) Atomic resolution description of an archaeal cell surface. The S-layer lattice is completed by pentameric defects (colored darker) (98). Panels C, D, E, F, and H are adapted from references 107, 67, 108, 109, and 98, respectively, under the Creative Commons Attribution 4.0 International License (https://creativecommons.org/licenses/by/4.0/).

The establishment of *Hfx. volcanii* as a model organism further accelerated the development of multidisciplinary methods over the past few decades (Table 1), making it a versatile platform for studying a wide array of archaeal biology. Research on *Hfx. volcanii* ranges from genetic information processing—such as DNA replication and repair, transcription, tRNA and rRNA maturation—to regulation, including post-translational modifications, CRISPR-Cas, cell signaling, as well as key aspects of cell physiology, such as cell shape, motility, biofilm formation, and adaptation to saline environment (Fig. 2). In this section, we will highlight selected discoveries where *Hfx. volcanii* studies have provided pioneering or unique insights into archaeal biology.

### Polyploidy

Oligoploid (up to 10 copies of the chromosome) and polyploid (more than 10 copies of the chromosome) genomes are common in prokaryotes (38, 111–115), particularly in haloarchaea, where all six analyzed species exhibit polyploidy (38, 116, 117). For example, *Hbt. salinarum* and *Hfx. volcanii* have about 25 copies and 18 copies of chromosomes, respectively, in their exponential phase, while the number decreases for both species in their stationary phase (38). Polyploidy is suggested to have multiple evolutionary advantages (118), including its roles in global gene dosage regulation and DNA repair. Achievement of those advantages often requires an efficient homologous recombination mechanism. Using *Hfx. volcanii*, Zerulla et al. demonstrated that both intracellular and extracellular DNA can serve as phosphate storage polymers in *Hfx. volcanii* (119). The number of *Hfx. volcanii* chromosomes decreased in phosphate-limiting media and increased in phosphate-rich media, suggesting that polyploidy serves as a mechanism for essential nutrient storage, independent of homologous recombination.

In addition, *Hfx. volcanii* also serves as an ideal model to study intermolecular gene conversion for equalization of multiple gene copies, which has only been studied in a few archaeal species, including *Hfx. volcanii* (120, 121), *Methanococcus maripaludis* (113), and *Haloferax mediterranei* (121). Dattani et al. demonstrated that *Hfx. volcanii* equalizes its multiple genome copies more efficiently compared to its close relative *Hfx. mediterranei,* likely due to its higher homologous recombination rate (121). This could also explain how *Hfx. volcanii* escapes Muller’s ratchet, a major disadvantage of polyploidy that can lead to the accumulation of deleterious mutations and, ultimately, species decline or extinction. Recently, Özer et al. constructed single-gene deletion strains of 24 *Hfx. volcanii* genes to assess their roles in gene conversion and equalization (122). Sixteen single-deletion strains showed severe defects in gene conversion, suggesting the existence of a sophisticated regulatory mechanism underlying gene conversion. The exact role of these components and their coordination in the process warrant further investigations.

### Origin of replication

Origins are nucleotide sequences that initiate DNA replication by recruiting replicative machineries. While bacteria only have one replication origin per chromosome (123), and eukaryotes, on the other extreme, contain a very high number of origins (124), the number of origins on an archaeal chromosome is highly variable, ranging from one in *Methanosarcina mazei* to four in *Pyrobaculum calidifontis* (125). The wild isolate strain of *Hfx. volcanii* (DS2) has three origins on its main chromosome (126), which raises intriguing questions regarding the role of each origin in maintaining polyploidy. An analysis of two of the origins, origins 1 and 2, shows their important yet surprisingly opposite roles in regulating genome copy number and cell fitness (127). This is consistent with the theory that the origins are obtained independently from different genetic sources and are only incorporated into the genome later as origins (126).

Another major finding centered around the essentiality of origins. Due to their roles in DNA replication, origins were thought to be indispensable for cells. Consistently, deletion mutants of all three origins in *Sulfolobus islandicus* could not be generated (128), while in *Haloferax mediterranei*, deleting all three origins triggers the activation of a dormant origin that could not be further deleted (129). Hence, it was very striking when Hawkins et al. showed that origin-less *Hfx. volcanii* not only had no growth defect but also managed to even grow faster than the wild type (126). No dormant origins could be identified in the deletion strain, and its DNA replication was shown to be driven by RadA-dependent homologous recombination, a replication strategy used by certain viruses.

*Hfx. volcanii* also possesses an unusually high number of origin elements compared to other archaea. Typically, an archaeal origin consists of a DNA region known as oriC and a gene (*orc*) encoding the initiator protein “ORC1/Cdc6”; therefore, the numbers of origins and *orc* genes are equivalent. However, haloarchaea deviate from this pattern, exhibiting more *orc* genes than origins. This discrepancy is particularly striking in *Hfx. volcanii*, which has six origins but 16 *orc* genes (130). Intriguingly, these ORC proteins vary in the number of chromosomes they affect and the impact they have on the copy number, underscoring a complex regulatory network for genome copy number in this organism.

### Mating

The transfer of genetic information occurs in all domains of life through mechanisms such as transformation, conjugation, and mating. In archaea, the first genetic transfer system was discovered in *Hfx. volcanii* in 1985, where researchers generated prototrophic recombinant cells by mixing two auxotrophic mutant strains (48). This process, referred to as mating, is resistant to DNase treatment and involves bidirectional DNA transfer between parental cells via intercellular bridges (48, 131). It is interesting that this DNA transfer also happens between *Hfx. volcanii* and *Hfx. mediterranei*, two species that share an average of 86.6% sequence identity (132). Unlike the high recombination barriers observed between bacterial species, the interspecies mating efficiency between the two species only decreased by less than one order of magnitude compared to intraspecies mating of *Hfx. volcanii*. This finding raises important questions about archaeal speciation in the context of frequent interspecies recombination, suggesting the existence of yet-to-be-discovered recombination barriers that facilitate speciation. Previous studies in *Hfx. volcanii* have identified several factors affecting mating efficiency, including archaeosortases, biofilm formation, protein glycosylation, CRISPR-Cas, and restriction-modification systems (133–137). Yet, our understanding of the molecular mechanisms underlying key events, such as mating initiation, cell fusion, and DNA exchange remains fragmented. A recent study by Makkay et al. shed new insights into the molecular mechanisms by using transcriptomics to analyze differentially expressed genes at various stages of mating in *Hfx. volcanii* (138). Their findings not only show that mating is immediately triggered upon cell-cell contact but also highlight several groups of candidate genes that may act as key components in archaeal mating, such as glycosylation genes, DedA/SNARE genes, and CetZ/FtsZ. Sivabalasarma et al. contributed to our understanding of mating from a more macroscopic perspective (108). Using electron cryo-tomography and fluorescence microscopy, they reported that the intercellular bridges between *Hfx. volcanii* cells are enveloped by a surface (S) layer (Fig. 2E). Moreover, macromolecular structures like ribosomes and thin filamentous helical structures were visualized within the bridges, suggesting that the bridges also facilitate the transport of cellular components, in addition to DNA.

### Cell division, shape transition, and multicellularity

The first tubulin homolog characterized in any archaeon was FtsZ in *Hfx. volcanii*, where it was shown to play a central role in cytokinesis (139). *Hfx. volcanii* possesses two FtsZ homologs: FtsZ1 appears to scaffold the division ring, while FtsZ2 is essential for initiating constriction (140). Over the past two decades, research has uncovered additional components of the archaeal division machinery. A SepF homolog was found to anchor the Z-ring to the membrane (141), similar to its function in bacteria, and PRC-barrel domain proteins (CdpB1 and CdpB2) further stabilize divisome organization, with their depletion causing severe division defects (142, 143). Additionally, the transcriptional regulator CdrS integrates metabolic and division signals, regulating key cell division genes (144).

Beyond division, *Hfx. volcanii* undergoes dynamic shape transitions between disk and rod morphologies, which profoundly impact motility and cellular function. Recent interdisciplinary studies led to the identification of numerous cytoskeletal proteins involved in these transitions. CetZ1, an archaea-specific tubulin homolog distinct from the FtsZ family, localizes along the cell periphery and mid-cell, facilitating the transition from a non-motile disk to a motile rod shape (145) and the proper positioning of motility structures such as the archaellum (146). In contrast, the actin-like volactin forms intracellular filaments that facilitate the transition from rod to disk morphology (Fig. 2F) (109). Additionally, halofilins, archaeal homologs of bactofilins, have recently been shown to contribute to curvature-dependent morphogenesis (147). Halofilin A accumulates at positive membrane curvatures in rods, and halofilin B localizes at negative curvatures in disks. Their absence results in morphological defects and mispositioning of the future division plane, indicating their crucial roles in structural integrity. Additionally, the recent determination of the first atomic structure of a complete and native *Hfx. volcanii* S layer has revealed how its local organization adapts to accommodate variations in cell envelope curvature, highlighting the intricate relationship between cell shape and envelope architecture (98).

Recently, Rados et al. discovered a novel multicellular developmental program in *Hfx. volcanii* triggered by compression (47). These multicellular clones exhibit characteristics distinct from their typical unicellular lifestyle and similar to animal tissues (Fig. 2B). Through RNA-seq and CRISPRi libraries, they identified a set of proteins involved in mechanosensing and cell junction formation, including actin and components of the N-glycosylation pathway. Together, these findings establish *Hfx. volcanii* as a key model for studying archaeal cytoskeletal organization, cell division, morphogenesis, and multicellular systems.

### Post-translational modifications

Post-translational modifications, such as glycosylation, phosphorylation, methylation, acetylation, and lipidation, are reported to be widespread in archaea and essential for the adaptation to extreme environments (148). For example, initially believed to be unique to eukaryotes, the glycosylation of the S-layer glycoprotein (SLG) in *Hbt. salinarum* (36) was the first identified glycosylated protein in archaea, followed by the SLG in *Hfx. volcanii* (37). In a series of studies focusing on the highly abundant SLG in *Hfx. volcanii*, researchers identified three distinct *N*-glycans and one O-glycan (37, 149, 150). Benefiting from available genetic tools, the biogenesis pathways for two of those *N*-glycans have been characterized in detail, rendering *Hfx. volcanii* the only archaeon so far for which two independent *N*-glycosylation pathways have been described (151, 152). In addition, the importance of this modification was shown for a variety of cellular processes, including motility (153), biofilm formation (154), mating (136), S-layer function (155), and cell shape (Fig. 2) (156). Recent advancements in mass spectrometry have enabled an in-depth glycoproteomic analysis of *Hfx. volcanii*, identifying the largest archaeal glycoproteome described to date (156). Similarly, in addition to genetic analyses identifying essential acetyltransferases and deacetylases (157), proteomics revealed an extensive lysine acetylome in *Hfx. volcanii* and provided insights into its role in oxidative stress responses (158).

Another significant discovery is the characterization of ubiquitin-like small archaeal modifier proteins (SAMPs) in *Hfx. volcanii*. Ubiquitin-like proteins (Ubl), found in eukaryotes and bacteria, share a similar structure and sequence motif with ubiquitin (Ub) and can attach to proteins and lipids or aid in sulfur incorporation into biomolecules (159). It was long known that archaea have Ubl, but their exact functions remained unclear until small archaeal modifier proteins in *Hfx. volcanii*, SAMP1–SAMP3, were detected to form protein conjugates (160, 161). This protein conjugation system contains fewer ubiquitin-activating enzymes than eukaryotic systems, representing a more streamlined and potentially more evolutionarily ancient mechanism for protein conjugation. Work by Miranda et al. revealed that the *Haloferax* Ubl proteins are also involved in sulfur metabolism (162). Further studies identified additional proteins involved in SAMPylation and the ubiquitin-like proteasome system (Fig. 2G), providing new insights into the underlying molecular mechanisms and the evolution of the Ub/Ubl system (163–166).

### Protein secretion and surface anchoring

In addition, *in vivo* studies of *Hfx. volcanii* have provided unique insights into the biogenesis of surface proteins in archaea. It was revealed that the archaeal general Sec machinery closely resembles the eukaryotic pore, with homologs to bacterial accessory proteins (SecD and SecF) aiding in protein transport (23, 167, 168). Moreover, it was first demonstrated in *Hfx. volcanii* that archaea utilize the twin-arginine transport (Tat) pathway to secrete folded proteins, in contrast to the Sec pathway, which primarily transports unfolded proteins (55, 169, 170). Bioinformatic analyses predicted that haloarchaea rely more extensively on the Tat pathway for secreting surface proteins than other archaeal species. Since haloarchaea, including *Hfx. volcanii*, maintain osmotic balance by accumulating equimolar concentrations of salt within the cytoplasm—a strategy known as “salt-in”—their preference for the Tat pathway may be an adaptation to the saline cytoplasm, allowing post-translationally modified proteins to fold before secretion and preventing precipitation (55, 171). Folding in the cytoplasm may also prevent co-translationally transported proteins from precipitating, as the high-salt external environment lacks ATP-dependent chaperones that assist in proper folding. The diverse range of Tat substrates in haloarchaea also led to the development of the first Tat substrate prediction tool for both archaea and bacteria (55, 172).

*Hfx. volcanii* has also been an ideal model for studying the lipidation of prokaryotic proteins. For example, proteins with a conserved motif known as lipobox have been shown to be N-terminally anchored to membrane lipids in bacteria. Although lipobox-containing proteins are predicted to be widespread in archaea, particularly in halobacterial species, no homologs of bacterial lipoprotein biogenesis components have been identified, possibly due to the differences in archaeal and bacterial membrane lipid composition. A recent study in *Hfx. volcanii* identified two paralogous proteins, AliA and AliB, as crucial for archaeal lipoprotein lipidation and revealed their importance in cell growth, motility, and cell shape (173). Studies on *Hfx. volcanii* also uncovered the first prokaryotic pathway for C-terminal lipid anchoring of surface proteins, including the S-layer glycoprotein, mediated by archaeosortase, along with PssA and PssD (133, 174, 175). This pathway is also predicted to be responsible for anchoring multiple surface proteins in many other euryarchaeal species (133). Furthermore, single-cell microscopy in *Hfx. volcanii* demonstrated that S-layer biosynthesis occurs at the division site, linking cell wall growth to cell division (175).

### Small proteins

Small proteins, defined here as proteins smaller than 70 amino acids, have historically been underexplored due to challenges in their detection. Yet, recent technological advancements have significantly improved their identification, revealing their wide prevalence across domains and importance in cell physiology (176, 177). Weidenbach et al. provided a comprehensive review of archaeal small protein studies (178), highlighting the relatively high number of identified and characterized small proteins in *Hfx volcanii* compared to other archaea. Among the ~400 small proteins in *Hfx. volcanii*, 49 contain at least two C(*P*)XCG motifs and are therefore predicted as zinc finger proteins (179). As part of the Priority Program “Small Proteins in Prokaryotes: an Unexplored World,” the Soppa group generated more than 30 single-deletion strains of those putative zinc finger protein-encoding genes (180). Those mutant strains exhibited distinct phenotypes compared to wild type under at least one condition, including differences in growth, swarming, and biofilm formation (180), underscoring the functional importance of these small proteins in archaeal physiology. Further investigations into 14 purified C(*P*)XCG proteins suggested that they can be metal-free or bind zinc or other metals, offering new insights into the functional variability of zinc-finger-containing proteins (179). Additionally, Hadjeras et al. from the Marchfelder group recently combined small protein-optimized mass spectrometry with ribosome profiling and identified a significantly expanded *Hfx. volcanii* small proteome (80), laying a solid foundation for future research on small proteins in archaea.

### RNA biology

When the first small regulatory RNA (sRNA) was identified in *Escherichia coli* (181, 182), it was thought to be a rare exception. Yet, the essential role of sRNAs in regulation has been repetitively demonstrated across the domains of life in the past 40 years (183). *Hfx. volcanii* is one of the earliest archaeal species used for sRNA studies, which has a high abundance of sRNAs as revealed by RNomics (184). The same study demonstrated that two sRNAs are important for cell resistance to high temperature and low salt, providing the first insight into the functions of archaeal sRNAs, beyond the snoRNAs involved as guide RNA in tRNA and rRNA modification (184). *Hfx. volcanii* is also among the earliest ones analyzed by RNA-seq for small RNAs identification. In total, 145 intergenic sRNAs and 45 antisense sRNAs were identified (185). Moreover, this RNA-seq analysis also revealed a high concentration of tRNA-derived fragments, a new type of sRNA first identified in humans and not yet identified in archaea back then (185). This high-confidence inventory of *Hfx. volcanii* sRNAs laid a firm foundation for accelerated characterization of their functions and the underlying mechanisms of action in the following years, revealing their roles in zinc transport and diverse stress response processes (76, 186–189).

*Hfx. volcanii*, despite its lower amount of tRNA and rRNA modifications compared to other archaea, is one of the few organisms for which the nearly complete set of tRNA modifications has been experimentally determined (190–193). Building on this foundation, research has increasingly focused on elucidating the biosynthesis pathways underlying these modifications. Using comparative genomics, Grosjean et al. predicted almost all genes coding for tRNA and rRNA modification enzymes in *Hfx. volcanii* (190). Subsequent studies characterized multiple tRNA-modifying enzymes and their interactions, including methyltransferases (Trm9 and TrmY) (194, 195), the methyltransferase activator Trm112 (196), the pseudouridine synthase TruA (197), the cytidine acetyltransferase TmcA (74), and several enzymes involved in the synthesis of archaeosine (73, 74, 198–201), an archaea-specific tRNA modification. Similarly, advances in sequencing have accelerated discoveries in the biogenesis pathway of rRNA modifications (79) and their roles in cellular fitness (202), further highlighting the intricate regulatory roles of RNA modifications in *Hfx. volcanii*.

### Metabolism

The metabolism of *Hfx. volcanii* has been extensively studied, greatly facilitating the development of genetic tools. For example, several enzymes involved in pyrimidine and amino acid metabolism, such as *pyrE2*, *leuB*, and *trpA*, have been developed into widely used selectable markers (50, 61). Such studies have also uncovered unique aspects of archaeal metabolism. For instance, the archaeal mevalonate pathway for isoprenoid biosynthesis remained unresolved for a long time until the discovery of an alternative mevalonate pathway in *Hfx volcanii* (203). Similarly, the conversion of glucose to ribulose-5-phosphate was long unresolved until the identification of the archaea-specific enzyme Azf in *Hfx. volcanii* (204). A distinguishing feature of *Hfx. volcanii* compared to *Hbt. salinarum* is its ability to grow on single-carbon compounds. Leveraging this trait, researchers have extensively investigated acetate metabolism (205) and sugar degradation pathways, including those for D-xylose, fructose, L-arabinose, L-rhamnose, and D-galactose (206–210). Additionally, thiamine biosynthesis in *Hfx. volcanii* has been shown to follow a hybrid pathway resembling both bacterial and eukaryotic components (211, 212). Furthermore, the biosynthesis of the second messenger cyclic di-AMP was shown to be mediated by the enzyme DacZ (213). Collectively, these findings highlight the distinctive metabolic capabilities of *Hfx. volcanii* and expand our understanding of archaeal metabolism.

## FUTURE PERSPECTIVE AND EMERGING DIRECTIONS

### Building on success: *Hfx. volcanii* as a model for advancing archaeal biology

Despite the wealth of knowledge gained through *Hfx. volcanii* studies, many fundamental questions in archaeal biology remain unanswered. One particularly intriguing aspect is *Hfx. volcanii*’s polyploidy, which raises intriguing questions regarding the regulation of DNA replication, repair, and segregation across multiple chromosome copies. Understanding how gene copy number influences transcriptional regulation, metabolic adaptation, and stress responses could provide valuable insights into polyploidy as a strategy for survival in extreme environments. In addition to DNA-level regulation, RNA-based mechanisms also play a crucial role in archaeal adaptability. Further investigation of the biogenesis of archaeal RNA modifications and their physiological functions, non-coding RNA functions, and small RNA-mediated regulation could offer new perspectives on evolutionary innovation and the diversification of regulatory networks in archaea.

The interplay between translation, protein folding, and post-translational modifications remains a critical area of research. For example, while the archaeal proteasome is known to play a key role in protein quality control and has different substrates under various environmental stresses (110, 214–216), several questions remain about how substrate selection occurs and the regulatory mechanisms that control proteasome activity in response to cellular needs. In addition to protein turnover, the dynamics of protein transport systems also remain largely unresolved in *Hfx. volcanii*. The Tat and Sec pathways are known to transport proteins across the cytoplasmic membrane, but substrate specificity and regulation during different growth conditions or stress responses are not fully characterized. Addressing these gaps could provide critical insights into the evolution of protein trafficking and its adaptation to extreme environments. *Hfx. volcanii* also offers a unique opportunity to study the roles, regulation, and crosstalk of post-translational modifications, such as glycosylation, acetylation, and SAMPylation. While the corresponding biosynthesis pathways have been partially characterized (217–219) and proteomics studies have identified modified proteins (156, 158, 220), the functions of many modified proteins remain unknown. Of particular interest is the regulation of protein glycosylation, as multiple glycosylation pathways have been shown to modify the same proteins (156, 221). Additionally, glycosidases that cleave off glycans and may help in dynamic adaptations to changing conditions have not been identified yet.

Furthermore, key processes essential to archaeal cell physiology remain to be fully elucidated. For example, the separation of the membrane during cell division continues to be an active area of research. While proteins such as CdpA, FtsZ1, and FtsZ2 have been identified as essential components for normal cell division in *Hfx. volcanii* (140, 144), further structure-function studies are needed to fully understand the roles of each component and how they coordinate functionally. These studies can also aid in identifying new components involved in cell division. New insights into the mechanisms underlying archaeal cell shape will also be exciting. For example, how do the structural proteins that shape *Hfx. volcanii*’s pleomorphic cells coordinate with cell cycle progression, and how are these processes modulated in response to environmental challenges? Another adaptation to changing environmental conditions, the formation and dispersal of archaeal biofilms, remains largely enigmatic as well, especially concerning its regulation at the molecular level. Investigating the signals and genetic circuits involved could unveil how archaeal communities adapt and thrive under diverse conditions.

With the growing interest in archaeal biology and the expanding toolkit available for *Hfx. volcanii*, these future directions promise to significantly advance our understanding of the archaeal domain. By exploring these fundamental aspects of archaea, we can gain new insights into the evolutionary relationships between archaeal systems and their bacterial and eukaryotic counterparts, answering broader questions about the origins of life and the evolution of complex cellular systems.

### Emerging trends in omics techniques

The continued expansion and refinement of omics approaches and databases hold tremendous potential for advancing our understanding of *Hfx. volcanii* and archaeal biology. For example, incorporating detailed functional predictions of non-coding RNAs, regulatory elements, and operon structures into the *Hfx. volcanii* genome annotations will provide critical insights into gene regulation and genome organization. Enhancing these annotations with experimental data, such as CRISPR-based gene knockdowns and transcriptomic profiling under diverse conditions, could uncover novel regulatory networks and stress response pathways.

Proteomics is well suited for the large-scale analysis of post-translational modifications, protein transport, protein-protein interactions, and proteasome-mediated protein turnover. Advancements, particularly in quantifying dynamic changes in the abundances of proteins, their modifications, and interactions will be pivotal for elucidating the molecular basis for various cellular processes in archaea. Integrating these proteomics results with systems biology approaches could identify targets for engineering *Hfx. volcanii* as a production host for extremophilic enzymes and other high-value biomolecules. Additionally, refined metabolomic analyses could help map unique archaeal metabolic pathways, enabling metabolic engineering strategies to harness *Hfx. volcanii* for industrial processes such as bioremediation or biofuel production.

To support these endeavors, a stringent use of existing databases, such as NCBI (222) and PRIDE (223), along with the development of tools—such as those within the ArcPP (59)—that integrate both existing and continuously submitted data, is essential and should be tailored specifically to archaeal biology. These platforms could integrate multiomics data, molecular regulation networks, protein structures, and interaction maps, providing researchers with a comprehensive resource for hypothesis generation and experimental design. Advanced machine learning-based models could not just provide input for such databases (e.g., structure and interaction predictions by AlphaFold [224]) but could also be trained on the integrated wealth of information, thereby facilitating the further prediction of enzyme functions, regulatory motifs, and metabolic fluxes, accelerating the pace of discovery and application. Notably, archaea are currently underrepresented in many databases that have been used for training prediction tools (e.g., <3% of PDB structures used to train AlphaFold 3 [225] are of archaeal origin; similarly, only about 1% of known signal peptide cleavage sites used to train SignalP 6.0 [83] are from archaeal proteins). Thus, an improved data foundation, advances in domain adaptation, and experimental validation of predictions are needed.

By leveraging these omics technologies and databases, *Hfx. volcanii* will continue serving as a premier model not only for dissecting archaeal molecular biology but also for understanding archaea at a systems biology level, both of which form the foundation for advancing *Hfx. volcanii* as a robust platform for biotechnological innovation. These efforts will bridge fundamental research with practical applications, highlighting the untapped potential of archaea in addressing global challenges.

### From lab to industry: harnessing *Hfx. volcanii* for biotechnological innovations

The same features that make *Hfx. volcanii* an ideal model for basic research also contribute to its promise in biotechnology. Its ease of cultivation and natural competence for transformation enable rapid genetic modifications, facilitating the engineering of metabolic pathways and the production of recombinant proteins. The development of new plasmids with constitutive and inducible promoters has allowed for further optimized expression of economically valuable biomolecules (65), including enzymes highly tolerant to salt and potentially other extreme conditions (226). Additionally, the high-salt requirement of *Hfx. volcanii* significantly reduces the risk of contamination in large-scale cultures. The recent development of immobilized *Hfx. volcanii* in alginate beads further addresses challenges such as biofilm formation and machine corrosion, enabling the generation of robust whole-cell expression systems (Fig. 2D) (67, 227, 228). These characteristics make *Hfx. volcanii* highly suitable for industrial applications, including chemical synthesis, food production, and bioremediation. For instance, *Hfx. volcanii* has been engineered for high-level synthesis and secretion of endogenous laccases, a family of versatile oxidase enzymes crucial for pollutant degradation, bio-based polymer synthesis, and environmental cleanup (229). Due to their extremophile origin, these laccases are found to be valuable for industrial processes that require stability under extreme ionic conditions, such as detergent manufacturing and bioremediation in saline environments.

C_50_ carotenoid, a non-enzymatic biomolecule produced by *Hfx. volcanii*, is another fascinating compound with significant potential in biotechnology. Known for its antioxidant properties, C_50_ carotenoid is particularly valuable in industries such as pharmaceuticals and cosmetics, where it can protect cells and tissues from oxidative damage, offering potential benefits in anti-aging and skin care products (230, 231). Additionally, C_50_ carotenoid shows great promise in the development of durable materials for industries requiring resistance to harsh conditions, such as aerospace and marine applications, further underscoring its potential in biotechnology (232, 233).

*Hfx. volcanii* has shown great capabilities in the production of nanoparticles (NPs), a field with promising applications in biotechnology and nanotechnology (234). Recently, the synthesis of silver (Ag) and gold (Au) NPs by this model haloarchaeon has been reported. The gold nanoparticles exhibited anti-bacterial properties, indicating their potential in medical and environmental applications. Additionally, these gold nanoparticles were found to enhance the specificity of polymerase chain reactions, offering benefits for molecular biology research. Both Ag and Au NPs also displayed catalytic activity in the reduction of 4-nitrophenol when incubated with sodium borohydride, suggesting their utility in chemical processes.

Gas vesicles are air-filled nanostructures endogenously expressed by certain bacteria and haloarchaea, such as *Hbt*. salinarum. They confer buoyancy by trapping gas within their hollow structures (235, 236). Although *Hfx. volcanii* does not naturally produce gas vesicles, it can be engineered to heterologously express and study these proteinaceous nanostructures (237, 238). Moreover, gas vesicles can streamline downstream processing in a biotechnology setting by facilitating the purification of specific cellular products through flotation-based separation rather than centrifugation, leading to more cost-effective bioproduction systems (239, 240). Beyond their physical properties, gas vesicles have gained recognition for their utility as contrast agents in medical imaging, such as ultrasound and MRI (236). Engineering *Hfx. volcanii* to produce these structures opens avenues for their use as non-invasive biomarkers or as functionalized nanocarriers for drug delivery and other therapeutic applications.

These applications of *Hfx. volcanii* exemplify its remarkable versatility as both a model organism and a powerful biotechnological workhorse, bridging the gap between fundamental research and practical innovation. In recent years, focus has been directed toward studying the physiology of archaea with biotechnological potential and designing bioreactors and bioprocesses that increase productivity. These efforts are expected to further increase the growth rates and biomass production yields of archaea, making them even more powerful tools in biotechnological applications.

### Educational outreach and accessibility

*Hfx. volcanii* has emerged as an innovative and accessible tool for Science, Technology, Engineering, and Mathematics (STEM) education. Its high-salt growth requirements make it and other halophilic archaea exceptionally safe for classroom use, as these conditions inhibit the growth of pathogens and other potential contaminants. This eliminates the need for sterile equipment and allows for straightforward experimental setups that are ideal for teaching microbiology and cell biology, even in resource-limited settings.

At the high school level, *Hfx. volcanii* provides students with hands-on learning opportunities while addressing common barriers to microbiology experiments. For example, students can explore UV mutagenesis and photorepair by leveraging *Hfx. volcanii*’s innate ability to repair UV-induced DNA damage under light conditions. By exposing cultures to UV light and then incubating them in light and dark settings, students can directly compare growth, providing a clear introduction to DNA damage and repair mechanisms (241).

These same experiments are equally valuable at the undergraduate level, where they can be used to further explore the unique characteristics of archaea. The Kirby-Bauer assay, in particular, offers a great opportunity to introduce archaea into a commonly taught microbiology technique used to assess bacterial susceptibility to antibiotics. Students can use the Kirby-Bauer assay to study antibiotic resistance by observing growth patterns in the presence or absence of antibiotics. By comparing the response of *Hfx. volcanii* to antibiotics with that of bacteria, students gain deeper insights into the structural and physiological differences between these domains (242).

For more advanced inquiry-based lab courses, *Hfx. volcanii* also provides opportunities to perform genetic screens and experiments that investigate fundamental questions in microbiology. Students can identify novel components of motility systems or characterize unannotated genes, contributing to ongoing research in archaeal biology. These projects not only enhance students’ technical skills but have also led to peer-reviewed publications co-authored by undergraduates, providing authentic research experiences and promoting engagement with STEM fields (96, 107, 243, 244).

One of the exciting aspects of *Hfx. volcanii* in classrooms is the growing availability of online databases and resources. Resources like ArcPP and other archaeal-specific databases provide rich, interactive content for students. These resources allow students to explore genomic, proteomic, and metabolic data for *Hfx. volcanii* in an accessible and engaging way, promoting bioinformatics and computational biology skills. By integrating these databases into classroom experiments, educators can enhance students’ understanding of genomic research and its application to real-world science, even in remote or underresourced settings.

By integrating *Hfx. volcanii* into STEM education, educators can offer students equitable access to meaningful research experiences that inspire curiosity and scientific inquiry. This innovative approach underscores the value of archaea as models for both fundamental and applied science while nurturing the next generation of diverse, skilled, and inspired STEM leaders.
